# Oral Cells-On-Chip: Design, Modeling and Experimental Results

**DOI:** 10.3390/bioengineering9050218

**Published:** 2022-05-19

**Authors:** Hamed Osouli Tabrizi, Abbas Panahi, Saghi Forouhi, Deniz Sadighbayan, Fatemeh Soheili, Mohammad Reza Haji Hosseini Khani, Sebastian Magierowski, Ebrahim Ghafar-Zadeh

**Affiliations:** 1 Biologically Inspired Sensors and Actuators (BioSA) Laboratory, Lassonde School of Engineering, York University, Toronto, ON M3J 1P3, Canada; htabrizi@cse.yorku.ca (H.O.T.); panahiyu@yorku.ca (A.P.); sforouhi@yorku.ca (S.F.); denizsdg@yorku.ca (D.S.); fsoheili@yorku.ca (F.S.); hosseinkhani.farhad@gmail.com (M.R.H.H.K.); 2 Department of Electrical Engineering and Computer Science, Lassonde School of Engineering, York University, Toronto, ON M3J 1P3, Canada; magiero@eecs.yorku.ca; 3 Department of Biology, Faculty of Science, York University, Toronto, ON M3J 1P3, Canada

**Keywords:** capacitive sensor, complementary metal-oxide-semiconductor, oral epithelial cells, oral neutrophils, saliva

## Abstract

Recent advances in periodontal studies have attracted the attention of researchers to the relation between oral cells and gum diseases, which is a real threat to overall human health. Among various microfabrication technologies, Complementary Metal Oxide Semiconductors (CMOSs) enable the development of low-cost integrated sensors and circuits for rapid and accurate assessment of living cells that can be employed for the early detection and control of periodontal diseases. This paper presents a CMOS capacitive sensing platform that can be considered as an alternative for the analysis of salivatory cells such as oral neutrophils. This platform consists of two sensing electrodes connected to a read-out capacitive circuitry designed and fabricated on the same chip using Austria Mikro Systeme (AMS) 0.35 µm CMOS process. A graphical user interface (GUI) was also developed to interact with the capacitive read-out system and the computer to monitor the capacitance changes due to the presence of saliva cells on top of the chip. Thanks to the wide input dynamic range (IDR) of more than 400 femto farad (fF) and high resolution of 416 atto farad (aF), the experimental and simulation results demonstrate the functionality and applicability of the proposed sensor for monitoring cells in a small volume of 1 µL saliva samples. As per these results, the hydrophilic adhesion of oral cells on the chip varies the capacitance of interdigitated electrodes (IDEs). These capacitance changes then give an assessment of the oral cells existing in the sample. In this paper, the simulation and experimental results set a new stage for emerging sensing platforms for testing oral samples.

## 1. Introduction

Recent periodontal research studies have found a correlation between gum diseases and other conditions such as diabetes, osteoporosis, and human immunodeficiency virus (HIV) [1,2,3]. It is also well documented that patients with severe osteoporosis will likely experience periodontal breakdown [4]. There are also reports regarding the increased populations of periodontal-related bacteria in patients with HIV [5,6]. Moreover, there is a high risk of heart diseases in people with unhealthy gums, which is a result of bacterial infection in the bloodstream [7,8]. In addition, recent studies show a link between the salivatory oral cells such as Oral Polymorphonuclear Neutrophils (oPMNs) and gum diseases [9,10,11]. Indeed, the quantification and analysis of oral cells can improve our understanding of the cellular activities in saliva and might deliver a clear picture of oral health. For example, it has been verified that the number of oPMNs in patients with periodontitis is 4 times higher in comparison to healthy individuals [12]. The presence of an excessive number of oPMNs might have a negative impact on the oral tissue’s integrity. The secretion of effective active mediators into the oral cavity may endanger oral homeostasis [13]. As a result of periodontitis and chronic activation of oral neutrophils, there might be damage to the periodontal connective tissue, which leads to the loss of attachment, alveolar bone, and tooth loss [14].

Another example is a link between the epithelial cells derived from oral cavities and oral health. As per recent studies, epithelial cells contain biomarkers for oral diseases such as oral cancer. These research findings further show the importance of oral cell analysis using advanced sensing technologies to improve our understanding of the role of salivary cells in the gum and other inflammatory diseases [15,16].

Until now, there has been no established sensing platform for analyzing oral cellular activities. Researchers employ standard methods such as fluorescence microscopy and flow cytometry [17]. These methods need specialized equipment with tens of thousands of dollars, a costly service contract, and trained staff to operate it. Therefore, their use is restricted to well-funded laboratories [18]. In this paper, we address the challenge of developing a sensing platform for analyzing oral cells using Complementary Metal-Oxide-Semiconductor (CMOS) technology. Among various sensing technologies, CMOS has offered great advantages for integrating a single small chip featuring millions of sensors with high signal-to-noise ratios (SNRs), low cost, and high speed [19]. To date, many types of CMOS biosensors have been developed by researchers for cell monitoring applications using various approaches such as optical [20], capacitive [21,22,23], impedimetric and multi-modal [24] techniques. In these works, cells are cultured on the chip surface, and the sensors work based on the adhesion of cells to the substrate due to the adsorption of cell surface protein on the substrate. They are mostly reported for cancer cells like human ovarian cancer cells [21], human lung carcinoma cells [22], breast cancer cells [20], and also bacterial cells such as *E. coli* [23]. In this paper, we investigate the advantages of the CMOS capacitive sensor for studying oral cells.

Capacitive sensors are one of the popular CMOS biosensors reported for precise measuring of the capacitance variations at the electrode-sample interface in different cellular assays. They can offer significant advantages for studying growth and other cellular activities [22,25]. In these applications, the cells are firmly attached to the surface through integrin-containing multi-protein structures [26]. However, there are some challenges to studying oral cells and doing research on them [27]. Unlike the other cells, oral cells have very low culture ability. Thus, they have less affinity to the surface even if they are in a culture medium [28,29,30]. Although researchers have made some efforts to culture oral cells in saliva-like medium, they have not been completely successful [27]. In many experiments for culturing neutrophils, the cells underwent apoptosis, died after less than an hour and their morphology had changed [27]. In most tests on periodontal diseases, normal saline was used to take samples and study saliva cells without culturing them. Oral cells should be studied in the material most similar to the saliva-like medium in order to keep them alive during the experiments [31]. These cells cannot be cultured in an in vitro environment because they have a short life span [32]. It is worth mentioning that since oral neutrophils are activated during their migration from blood circulation to the oral cavity, they cannot be divided anymore. Therefore, they are in the last stage of their life cycle, and their nucleus cannot make cytokines as well [32,33]. There are different types of cluster of differentiation (CD) markers on the surface of neutrophil cells that might be responsible for cell attachment. According to the literature, the essential CD markers accountable for cell attachment are Mac-1 (CD 11b and CD 18) [33,34,35,36]. These CD markers may increase the affinity of cells to the hydrophilic surface of the silicon oxide or aluminum oxide above the CMOS chip.

**Figure 1:**
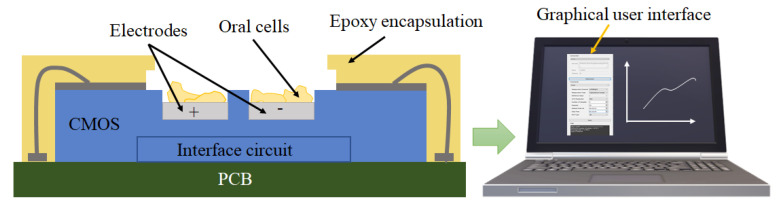
A CMOS-based capacitive sensor encapsulated with epoxy for oral cell monitoring.

This paper addresses the challenge of monitoring the hydrophilic adhesion using a CMOS capacitive sensing platform. As seen in Figure 1, the proposed platform includes a CMOS capacitive sensor featuring sensing electrodes and an interface circuit. The chip is encapsulated with epoxy with an opening for introducing the sample. This chip is connected to a computer through a printed circuit board (PCB) populated with off-chip components. This board provides the chip’s controlling signals and makes its output signal ready to send to the computer. A graphical user interface (GUI) is also used to help capture the output data in a user-friendly manner by employing interactive visual components of computer software.

The rest of the paper is organized as follows. Section 2 gives a brief overview of the related CMOS capacitive sensors. In Section 3, the materials and methods are presented. Section 4 demonstrates the implementation and experimental results. In Section 5, we discuss the practical considerations, and future works. Finally, our conclusions are drawn in Section 6.

## 2. Related Works

To date, many papers have reported the advantages of CMOS capacitive sensors for monitoring the growth or proliferation of living cells such as breast cancer cells [1], human lung carcinoma cells [2,3], or *E. coli* [4]. As seen in Table 1, for tracking the proliferation of MDA-MB-231 breast cancer cells, a capacitive sensor, with a resolution of 5 fF and passivated aluminum (Al) electrodes, was employed [34]. In another work, Senevirathna et al. reported the development of a capacitive sensor with a higher resolution of 14.4 aF for analyzing human ovarian cancer cells with passivated aluminum electrodes [21,35]. In another effort, Widdershoven et al. [25,36] used a capacitive sensor with gold nanoelectrodes with a radius of 90 nm and a resolution of 1 aF for imaging living cells such as BEAS, THP1, and MCF7 cancer cell lines. In addition to cancer cells, CMOS capacitive sensors have successfully been employed for bacteria detection. For instance, Couniot et al. proposed a CMOS capacitive sensor with Al/Al_2_O_3_ electrodes for the detection of *S. epidermidis* [37,38]. In another effort, Ghafar-Zadeh et al. used a technique called charge-based capacitance measurement (CBCM) for the development of a ∑∆ capacitance-to-digital converter (CDC) [23]. This sensor was employed for monitoring the growth of *E. coli* [23] on a passivated aluminum interdigitated electrode (IDE) with a window in between the fingers (Al/wPass). Although this sensor showed a high resolution of about 10 aF, its input dynamic range (IDR) was limited to less than 3 fF. Indeed, other reported capacitive sensors [21,25,34,35,36] have also demonstrated a low resolution or high sensitivity but low IDR. Thus they suffer from parasitic capacitances created due to the debris and other remnants above the capacitive sensor. Our group has addressed this challenge by developing a new core-CBCM interface circuitry with a wide IDR of 400 fF [39,40] and a resolution of about 450 aF. This circuit works based on a current-mode technique using an extended charge-based capacitance measurement (ECBCM) structure and a current-controlled oscillator (CCO), which gains the advantage of a calibration-free approach utilizing a bank of reference capacitors as briefly described in Section 3.4.1. In this paper, we employ this CMOS sensor for oral cell monitoring for the first time. For this purpose, we proposed a new electronic circuit board and graphical interface to read out the sensing data from the capacitive sensor chip and demonstrate it on the computer screen. This sensing platform enables the measurement of capacitance changes caused by oral cells. The experimental results agree with the proposed equivalent circuit and the Multiphysics simulation results discussed in Section 3.2. Furthermore, the sensing electrodes in this paper and other reported papers include metal or metal oxides demonstrating hydrophilic properties suitable for measuring the hydrophilic materials such as oral neutrophils, which can attach to the surface of these electrodes [41].

## 3. Materials and Methods

In this section, new modeling is presented for the analysis of the electrodes–analyte interface and the biological and chemical protocols used for the experiments are introduced. Section 3.1 discusses the electrical equivalent modeling of the interface, and Section 3.2 is dedicated to the simulations of the electrodes’ response using COMSOL software. The biological and chemical protocols are described in Section 3.3. Section 3.4 is dedicated to introducing the capacitive sensing platform.

### 3.1. Electrical Equivalent Modeling

In this section, the electrical equivalent modeling is explained for three states: (1) When there is no sample on the electrode; (2) When the sample droplet exists on the electrode; (3) After the evaporation of the droplet. Then, the modeling of the oral samples on the chip is discussed based on these three states.

#### 3.1.1. Electrical Equivalent Modeling without Sample

**Figure 2:**
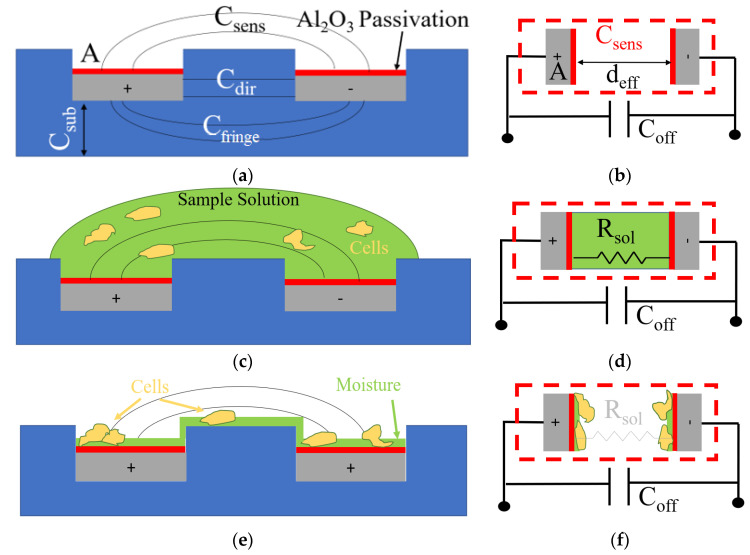
(**a**) Cross-section schematic of the implemented IDE electrode without sample; (**b**) Equivalent capacitance model without sample; (**c**) Cross-section schematic of the IDE electrode with the sample before the evaporation of the sample solution; (**d**) Equivalent capacitance model of the sample before the evaporation of the sample solution; (**e**) Cross-section schematic of the IDE electrode with the sample after the evaporation of the sample solution; (**f**) Equivalent capacitance model of the sample after the evaporation of the sample solution.

Figure 2 illustrates three distinct phases in the sample–electrode interface. A cross-sectional schematic of the electrodes implemented on the topmost metal layer of the CMOS chip is given on the left side of each row, whereas the proposed electrical equivalent model is shown on the right side of that row. One plate is always grounded in the implemented IDE topology, while the other plate is exposed to a pulsated voltage. In our topology, the voltage of the positive electrode reaches about one threshold voltage (0.6 V) less than *V*_dd_ (3.3 V) [39,40]. This voltage difference creates an electric field between the two plates of the IDEs with a fringe electric field passing above the electrodes, shown as *C_sens_*. As depicted in Figure 2a, other parasitic capacitances exist due to the underlying layers. *C_sub_* represents the offset to the top metal and the substrate. *C_sub_* can be calculated using the given value of the parasitic capacitance per area from the CMOS technology datasheets (1).

(1) $$C_{sub}=\alpha_{1}\times A+\alpha_{2}\times P$$

where *A* and *P* are the area and the perimeter of the implemented electrode, and $\alpha_{1}$ and $\alpha_{2}$ are process-dependent coefficients that are known for each fabrication technology. Another parasitic capacitance that is due to the direct electric field between the grounded plate and the voltage plate is shown as *C_dir_* and is a function of the area of the side of the plates that coincide with each other, as well as the electrode pitch, *d*, with the equation given in (2).

(2) $$C_{dir}=\varepsilon_{0}\;\frac{\varepsilon_{Sio_{2}\times P_{coincide}\times W_{th}\;}}{d}$$

where $\varepsilon_{0}$ is the vacuum permittivity, $\varepsilon_{SiO_{2}}$ denotes the relative permittivity of silicon oxide as the dielectric between the electrodes, $P_{coincide}$ stands for the perimeter of the plates that coincide with each other, $W_{th}$ represents the thickness of the electrodes, and d is the pitch of the electrodes. Considering the symmetry in the geometry of the IDEs, it can be assumed that *C_fringe_* is $\varepsilon_{SiO_{2}}$ times larger than *C_sens_*, as expressed in (3), since the dielectric material for *C_fringe_* is silicon oxide while it is air for *C_sens_* when there is no sample placed on the electrodes.

(3) $$C_{fringe}=\varepsilon_{Sio_{2}}\times C_{sens}$$

The value of the equivalent offset capacitance, *C_off_*, is thus equal to the addition of *C_sub_*, *C_dir_*, and *C_fringe_*, as given in (4).

(4) $$C_{off}=C_{sub}+C_{dir}+C_{fringe}$$

Figure 2b illustrates an equivalent electric model for the electrodes shown in Figure 2a, where *A* is the surface area of the electrodes and *d_eff_* is an adequate equivalent direct distance for the fringe distance. The strips shown in red represent the equal capacitance that forms due to the native aluminum oxide layer created on the aluminum electrodes because of its exposure to air. The thickness of the aluminum oxide layer naturally formed above the electrodes can reach less than 10 nm [44]. Since the relative dielectric constant of aluminum oxide is 9.5, this layer can create approximately 8.4 fF/µm^2^, which results in a high capacitance of about 59 pF considering that the dimensions of the implemented IDE with a surface area of 7056 µm^2^. The total equivalent capacitance for the electrodes without being exposed to any sample can be calculated using (5).

(5) $$C_{equivalant}=\varepsilon_{0}\;\frac{\varepsilon_{PL}\times \varepsilon_{Air}\times A}{\varepsilon_{PL}\times d_{eff-Air}+\varepsilon_{Air}\times \left(2d_{PL}\right)}+C_{off}$$

where $\varepsilon_{PL}$ stands for the relative permittivity of the aluminum oxide passivation layer, $\varepsilon_{Air}$ is the relative permittivity of air, and $d_{PL}$ represents the thickness of the passivation layer.

#### 3.1.2. Electrical Equivalent Modeling When the Sample Droplet Is Present

**Figure 3:**
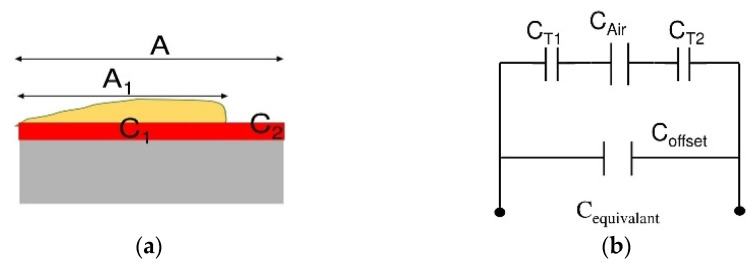
(**a**) Simplified model for the attachment of the cells on the electrodes after complete evaporation of the sample; (**b**) Equivalent circuit model.

Figure 3c demonstrates the electrodes when the sample solution is placed on top of the electrodes. As seen in the electric equivalent model given in Figure 2d, due to the conductivity of the sample solution that contains ions, a resistive path forms between the electrodes, *R*_sol_. In addition, a sizeable double-layer capacitance forms in the interface of the electrodes and the sample. The overall effect is saturating the sensor readings.

#### 3.1.3. Electrical Equivalent Modeling after Evaporation of the Droplet

Figure 2e demonstrates the phase in which the sample has evaporated while some moisture remains on the surface and the cells are deposited. In this phase, cell remnants play the role of a third dielectric in the equivalent capacitance of the passivation layer. Since the sample solution has evaporated, the value of resistance, *R*_sol_, is negligible. The amount of change in the total capacitance of the electrodes in the presence of cells compared to the capacitance of the electrodes with no sample is related to the percentage of the area of the electrode that is covered by cells, referred to as confluence, *conf*_*n*_ = *A*_1_/*A* (see Figure 3a). Figure 3b illustrates the equivalent electric circuit model for this phase. The deposition of cells on the electrode’s surface affects the passivation layer’s capacitance, shown as *C_T_*_1_ and *C_T_*_2_. Equations (6) and (7) describe *C_Tn_* and *C_equivalant_*, respectively.

(6) $$C_{Tn}=\varepsilon_{0}\times A\left(\;\frac{\varepsilon_{PL}\times \varepsilon_{cell}\times conf_{n}}{\varepsilon_{PL}\times d_{eff-cell}+\varepsilon_{cell}\times d_{PL}}+\frac{\varepsilon_{PL}\;\left(1-conf_{n}\right)}{d_{PL}}\right)\;n=1,\;2$$

(7) $$C_{equivalant}=C_{offset}+C_{T1}\left|\left|\;C_{Air}\right|\right|\;C_{T2}$$

where $\varepsilon_{cell}$ stands for the relative permittivity of the moist cells, and $d_{eff-cell}$ represents the average thickness of the sedimented cells. Equation (6) shows that the value of *C_Tn_* depends on the percentage of the coverage of cells on the electrodes, $conf_{n}$. Thus, an increase in the cell coverage leads to a rise in $C_{Tn}$. From (7), equivalent capacitance is equal to the combination of $C_{T1}$, $C_{T2}$*,* and $C_{Air}$. As a result, an increase in the measured *C_equivalent_* will be the representative of an increase in $C_{Tn}$. Therefore, the number of cells were present in the sample and were deposited on the electrode. This increase was observed in the simulation results discussed in Section 3.2.

#### 3.1.4. Oral Samples on the Chip

The addition of oral neutrophil samples on the chip mimics the three distinct states that were explained in Section 3.1.1, Section 3.1.2 and Section 3.1.3. In the beginning, to have a baseline, we perform a full reading of the sensor in phase one without introducing the sample. The readings reveal the *C_off_* + *C_sens_* mentioned in Figure 2b. Immediately after introducing the sample, the outputs become saturated due to the presence of the water droplet, as explained in detail in Section 3.1.2. After the water droplet evaporates, the model enters the third phase discussed in Section 3.1.3. In this phase, the sensor outputs reflect the amount of the change in the *C_sens_* due to the presence of wet oral cells on the electrodes. The experimental results are explained in detail in Section 4.2.

### 3.2. COMSOL Simulation

In this section, the capacitive response of the electrodes is simulated for two modes: (1) In the dry mode; and (2) when electrodes are exposed to cells in the water. In the wet mode, we also study the effect of the thick and thin layers of liquid. These simulations cover all three phases.

#### 3.2.1. Dry Mode

A COMSOL Multiphysics simulation was carried out to model the CMOS capacitive sensor’s static electrical response in the dry mode and when the sensor is subjected to a specific cell concentration. For this purpose, the sensor was modeled in the electrostatic module of COMSOL following the size and physical boundary conditions maintained in the fabricated sensor. In this module, the following Formulas (8) and (9), are solved numerically, which determine the sensor’s electrical potential for measuring the capacitance.

**Figure 4:**
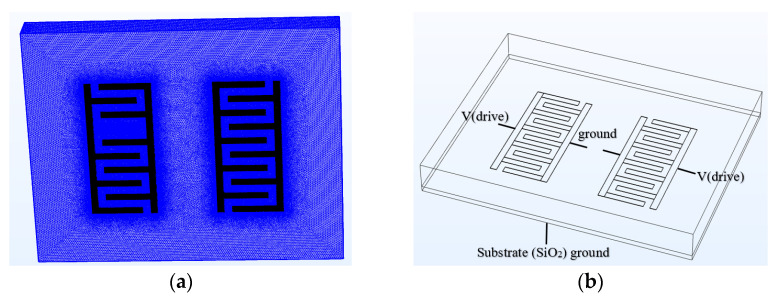
(**a**,**b**) Boundary conditions and meshing structure of the simulated sensor in COMSOL software.

(8) E=−∇V

(9) $$\nabla .\;\left(\varepsilon_{0}\varepsilon_{r}\right)=\rho_{v}$$

where *E* is the electric field; $\varepsilon_{0}$ denotes the vacuum permittivity; $\varepsilon_{r}$ is the relative permittivity; ρ stands for the electric charge density, and *V* is the electric voltage. To perform the simulation, the sensor was designed in the CAD module of COMSOL and then meshed to perform numerical simulation. A mesh analysis was performed to avoid the meshing effect on the result; consequently, the extra-fine mesh was utilized. The relative permittivity was used as the dielectric model. A schematic of the boundary conditions and the meshed structure are demonstrated in Figure 4.

**Figure 5:**
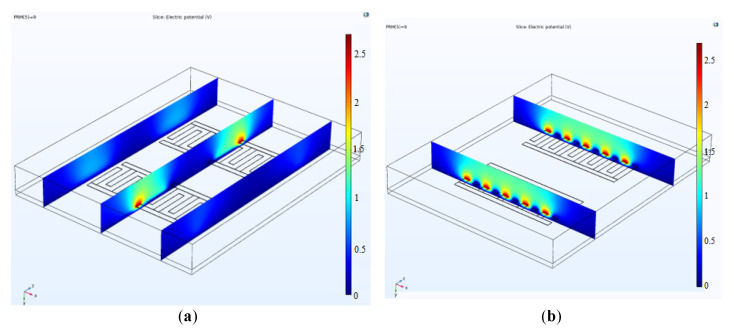
(**a**,**b**) The electrical potential counters of the sensor for a bias voltage of 2.7 Volts. The substrate is grounded.

Figure 5 demonstrates the electrical potential contours of the CMOS capacitive sensor when it is subjected to 2.7 V bias. The sensor was meshed with an extremely fine approximation to yield the best convergence. The sensor surrounding material is a thin layer of SiO_2_ identical to the CMOS fabrication materials. The simulation considers the capacitance due to the thin layer of SiO_2_ as the native material between the layers and the P-type silicon substrate of the sensor, which is electrically grounded. According to the simulation results, the equivalent capacitance for the sensor in the dry mode is around 117.415 fF. The capacitance is calculated by assuming that the sensor is surrounded by air.

#### 3.2.2. Exposing the Electrodes to Cellular Sample

**Figure 6:**
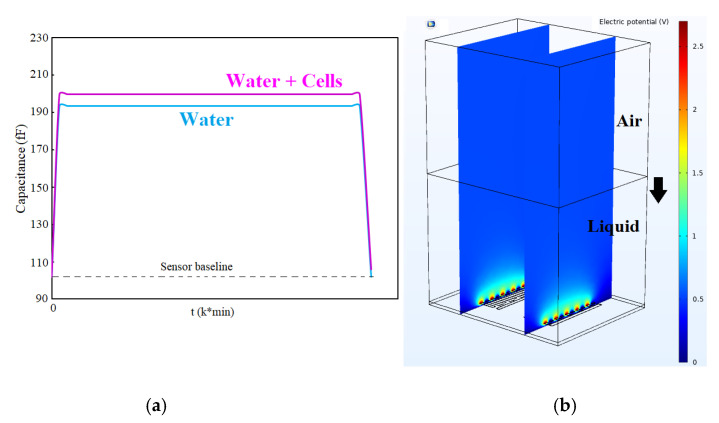
(**a**,**b**) COMSOL simulation showing the saturation of the sensor in liquids and returning to baseline.

According to the experimental evidence, the capacitance upsurges when exposed to a liquid volume. After complete evaporation of liquid on top of the sensor, the response gets back to the baseline considering the presence of cells. This also occurs when the medium encompasses cells or other components. Due to a change in the medium’s dielectric, the sensor response changes, and the cycle of rise-fall of capacitance would be different from the previous cycle with a different dielectric. To prove this observation and validate it, COMSOL Multiphysics was employed to simulate the cycle. Following the experiments, in modeling, we filled the top of the sensor with a thick liquid layer (here water) below the air layer as can be seen in Figure 6. Afterward, we changed the thickness of the water layer from 400 µm to 5 µm atop the sensor. This gradual decrease of the water layer thickness resembles the evaporation of the droplet. At the same time, the capacitance of each step was recorded. As seen in Figure 6, the sensor initially gets saturated, and then after complete evaporation of the droplet, it returns to the baseline. However, we expect different levels in the return cycle due to the existence of remnants of cells on top.

As shown in Figure 6, the electric potential on top of the sensor is decaying with the distance from the sensor surface. If the sensor environment is filled with a liquid (with a specific dielectric constant), the sensor response reaches a saturation level and constant level. When the liquid is evaporating, the cells’ concentration becomes more visible to the sensor by increasing the dielectric constant. Therefore, we can sense the mass of cells on the sensor surface. COMSOL simulation validates this idea that, with the capacitive sensor, we can measure the concentration of biological entities in the droplet by measuring the capacitance change during the sedimentation of cells on the sensor in a loop shown in Figure 6 and experiments.

### 3.3. Biological and Chemical Protocol

After introducing the employed materials and instruments in Section 3.3.1, the cleaning procedure is explained in Section 3.3.2. Then, the sample preparation protocol and control measurement techniques are described in Section 3.3.3 and Section 3.3.4, respectively.

#### 3.3.1. Materials and Instruments

In this work, various materials were used. These materials are 50 polypropylene conical tubes (Baxter, Toronto, ON, Canada); Sterile cell strainer: 40 μm nylon mesh (Millipore, Burlington, MA, USA); 11 μm nylon filters (Millipore, Burlington, MA, USA); 1.5 mL Eppendorf tubes (Fisherscience, Saint-Laurent, QC, Canada); 0.9% irrigation-grade sodium chloride solution (Baxter, Toronto, ON, Canada). We also employed several instruments, including an incubator (Heracell 150i, Thermo Fisher Scientific, Waltham, MA, USA); Lab Centrifuge (Sorvall ST 8, Thermo Scientific, Waltham, MA, USA); Hemocytometer (BLAUBRAND^®^ Neubauer Millipore Sigma, Burlington, MA, USA); Inverted and phase contract Microscope (isherbrand^TM^ Inverted Infinity, Phase contrast 10× and 20×, light splitter (100% or 20/80%), Fisher Scientific, Hampton, NH, USA).

#### 3.3.2. Cleaning Procedure

Although the goal in the field of CMOS-based biosensors is mass-production and designing single-use devices, it is important to wash and reuse them while they are still in the research stage. Because their cost is high when fabricated in limited quantity despite their cost-effectiveness in batch-production [45]. To test the functionality of the device, we gradually increase the cell concentration in each step to observe the differences between the device’s response to an increased number of cells. However, to have a precise measurement, it is crucial first to clean the surface and reuse it for the subsequent trial. We tried drop-casting and dipping in washing materials such as acetone, isopropanol alcohol (IPA), and ethanol; however, this method did not completely clean the surface. Therefore, we came up with using mechanical force to remove the cell residuals. Using a micrometric brush and droplets of Ethanol or IPA approximately solved the problem, and the surface was cleared from biological remnants.

#### 3.3.3. Sample Preparation

To prepare neutrophil samples, participants were advised to consume nothing but the water within 30 min before sample collection, rinse the oral cavity with 10 mL of tap water for 15 s, and discharge the rinsed fluid. The participants were asked to wait for 2 min before rinsing the oral cavity with 10 mL of a 0.9 percent *w/v* saltwater solution (normal saline) measured using the 15 mL falcon tube and eject the sample into the 50 mL falcon tube. After one wash, there was a 2-min wait time, and the same process was repeated five times. Then, the final sample was filtered gently via 40 μm filters to isolate the cells from the debris larger than 40 µm sizes. The tubes were centrifuged at 4 °C for 5 min with 2600 RPM and the supernatant was discarded until 1 mL of the sample remained in the falcon tube. Then, 1 mL of ultrapure water was added to the falcon tube and mixed with the cells. The related ethical approval for this research, including the human participants, was provided by York University.

#### 3.3.4. Control Measurement Technique

**Figure 7:**
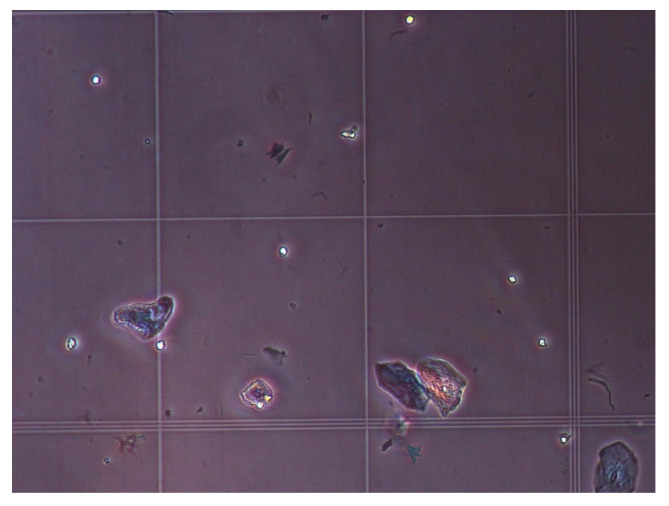
The microscopic images of the hemocytometer showing neutrophil and epithelial cells.

The hemocytometry cell counting technique was used to control the measurement results. At this step, the sample was thoroughly mixed. The hemocytometer was washed and cleaned with 70% (*v/v*) ethanol and allowed to dry. The coverslip was washed with 70% ethanol, allowed to dry, and placed on the hemocytometer counting chamber. A pipette was used to mix 10 μm of cells and 90 μm of Trypan Blue in a 1.5 mL Eppendorf tube. Then, 10 μL of cell suspension was added to Trypan Blue under the coverslip on a hemocytometer. After 2 min, the cells were counted. We estimated that the number of neutrophil cells in each 10 µL of saliva sample was about 7. The number of epithelial cells was around 13 in each microliter of the saliva sample. Figure 7 shows the microscopic images of the hemocytometer showing neutrophil and epithelial cells.

### 3.4. Capacitive Sensing Platform

**Figure 8:**
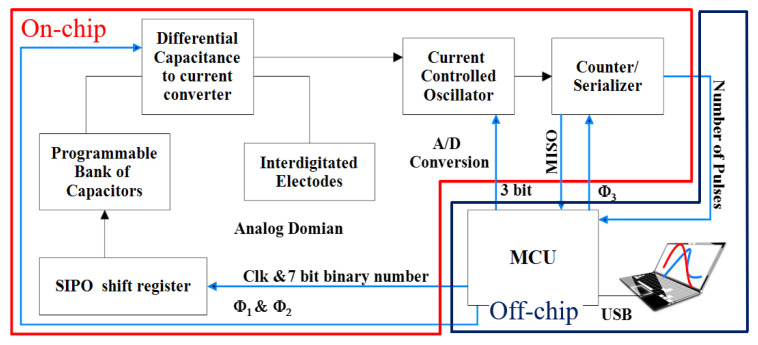
The block diagram of the proposed circuit and system.

The capacitance sensing platform is composed of on-chip and off-chip parts. A block diagram of the sensing platform is shown in Figure 8. On the CMOS chip, which was discussed in Section 3.4.1, the conversion of differential capacitance to digital output takes place in three modules. The chip requires external clock signals that were provided by utilizing a microcontroller circuit (based on an Arduino DUE board). The same microcontroller was used to read the digital output data of the chip through its serial peripheral interface (SPI) port. A PCB board was designed that hosts the chip and voltage supply (circuitry including voltage regulators) and allows the Arduino DUE board to be connected. This was explained in Section 3.4.2. A GUI was designed to fulfill the requirements for user preference settings and data visualization, which was explained in Section 3.4.3. The capacitance calculation algorithm was also described in Section 3.4.4.

#### 3.4.1. CMOS Capacitive Sensor

The implemented on-chip circuit is composed of a differential capacitance-to-current converter. The reference capacitance in this topology is a digitally programmable bank of capacitors that makes it possible to sweep the reference capacitance value. The output differential current of this block transfers to the CCO block. The oscillation frequency of the CCO is linearly related to the magnitude of the input current.

A counter is used to generate the digital output of the chip. The output of the chip is, as a result, the number of pulses, which is linearly related to the differential capacitance at the input stage of the capacitance of the IDE and the reference electrode (*C*_interdigitated_-*C*_reference_).

The value of this digitally programmable bank of reference capacitors can change from 200 fF up to 1270 fF with a step of 10 fF. This wide range of reference capacitors, instead of a single capacitor, enables measurement of a wide IDR. As a result, an additional axis, *C*_R_, was introduced to the measurements, changing the data from a single point to a 2D curve. The addition of a timestamp to the 2D curves results in the creation of 3D plots as a footprint of the sample (see Section 3.4.3).

#### 3.4.2. Off-Chip Circuit and System

**Figure 9:**
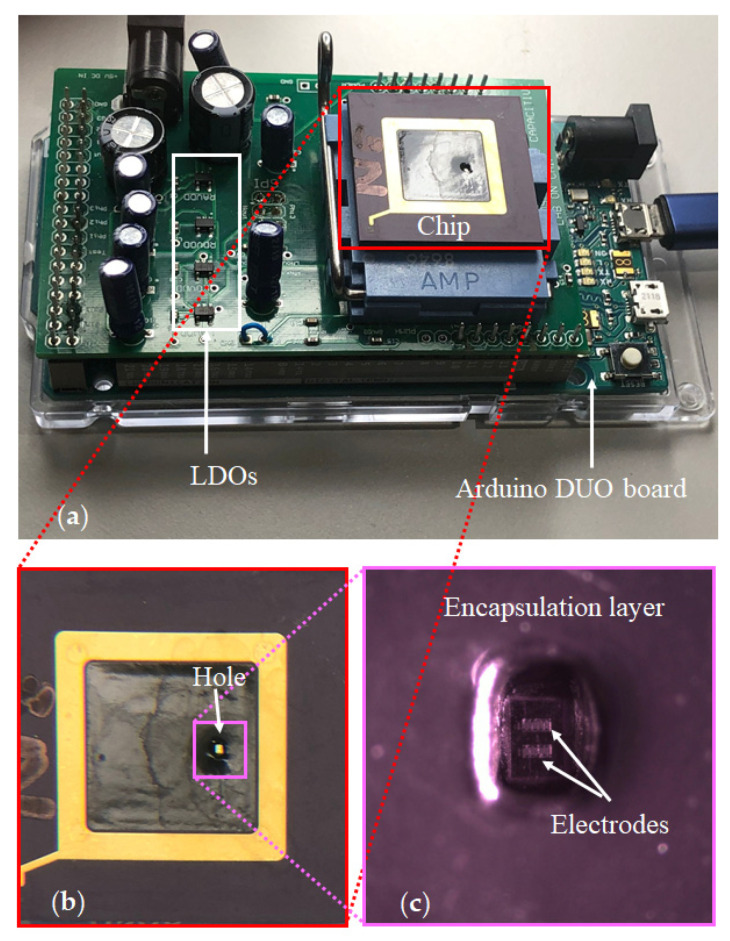
The testbench hardware: (**a**) An Arduino DUE microcontroller board together with a custom-made electronic board holding the chip; (**b**) The chip; (**c**) The microscopic image of the two electrodes and the surrounding encapsulation layer with a hole for the non-passivated sensing electrodes.

An Arduino DUE microcontroller board together with a custom-made electronic board that holds the chip and the required circuitry for voltages and the clock pulses are the testbench hardware, which is shown in Figure 9. Pulse Width Modulation (PWM) clocks with a frequency of 66.6 kHz are generated in channels 0 and 1 of the microcontroller with a duty cycle of 4/15 and 2/15 where they form in a non-overlapping fashion, and a 1 MHz clock with a duty cycle of 1/2 is generated in channel 3, which is used as the clock for the SPI port. A 2-to-1 multiplexer was also used to switch between the outputs of the right and left electrodes. The details of the circuitry can be seen in Appendix A.

#### 3.4.3. GUI Development

To practically utilize the chip, a dedicated testbench including both hardware and software was developed. The chip requires two sets of supply voltages for digital and analog parts and a reference voltage. The supply voltages are fed through Texas instruments low dropout voltage regulators TPS75901KTTR while adjusting the output to 3.3 V. Considering that we separated the left and right circuits, four voltage regulators in total were used. The CCO requires a 1.85 V reference that was supplied through the NCP705MTADJTCG adjustable output regulator from the ON Semiconductor. Required clocking signals were generated using an Arduino DUE board, and the SPI port of the board was used to capture the digital output data.

**Figure 10:**
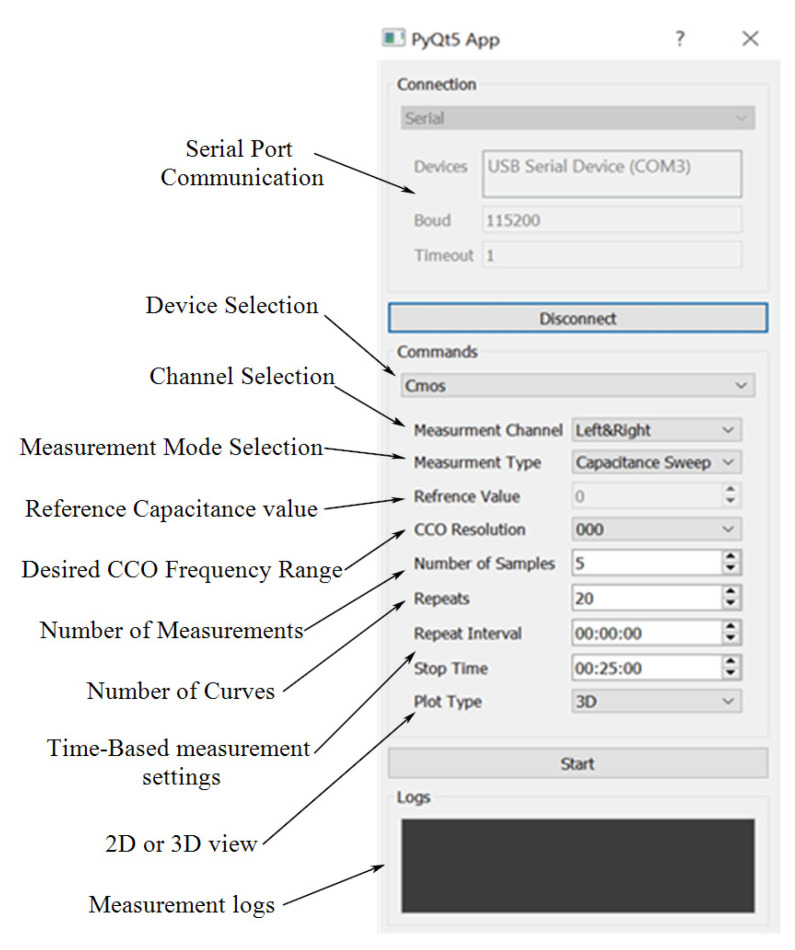
A snapshot of the GUI.

**Figure 11:**
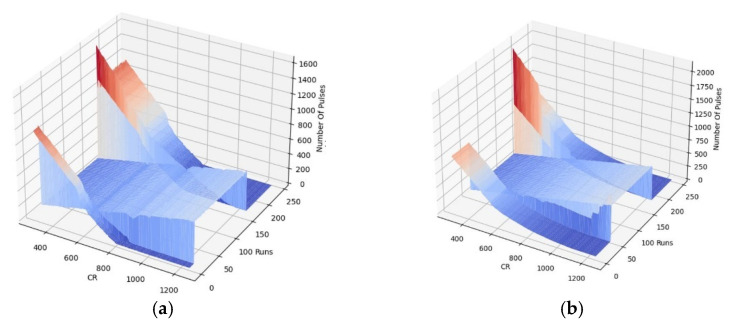
The visualized data captured by the GUI in real-time: (**a**) for the left IDE, (**b**) for the right IDE.

**Figure 12:**
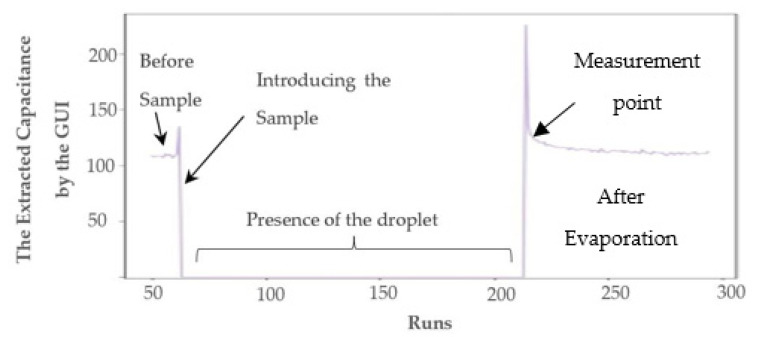
The extracted capacitance variations versus time.

The use of a testbench without a GUI is not very practical and user-friendly. As a result, a GUI was implemented in Python, enabling the users to configure their required set of experiments as well as perform data visualization. Figure 10 illustrates a snapshot of the GUI. Users can select capturing data from right, left, or both channels as well as select obtaining curves when the reference bank of the capacitor’s values are swept in the whole range or measured at a single point by determining the reference point value. In addition, by determining the number of samples, multiple measurements will be performed at a single reference point that can be averaged for better noise immunity. Users can set the GUI to obtain as many curves as desired or set the time-based measurement settings for the time interval of data acquisition and the experiment time. The visualized data is a three-dimensional (3D) curve with the chip’s output (number of pulses) on the z-axis, the values of the reference capacitance on the x-axis, and time on the y-axis. The GUI also extracts the value of capacitance from the captured data (see Section 3.4.4). A snapshot of the visualized data for both right and left channels is shown in Figure 11a,b, respectively. The variations of the extracted value of capacitance during the time are depicted in Figure 12.

**Figure 13:**
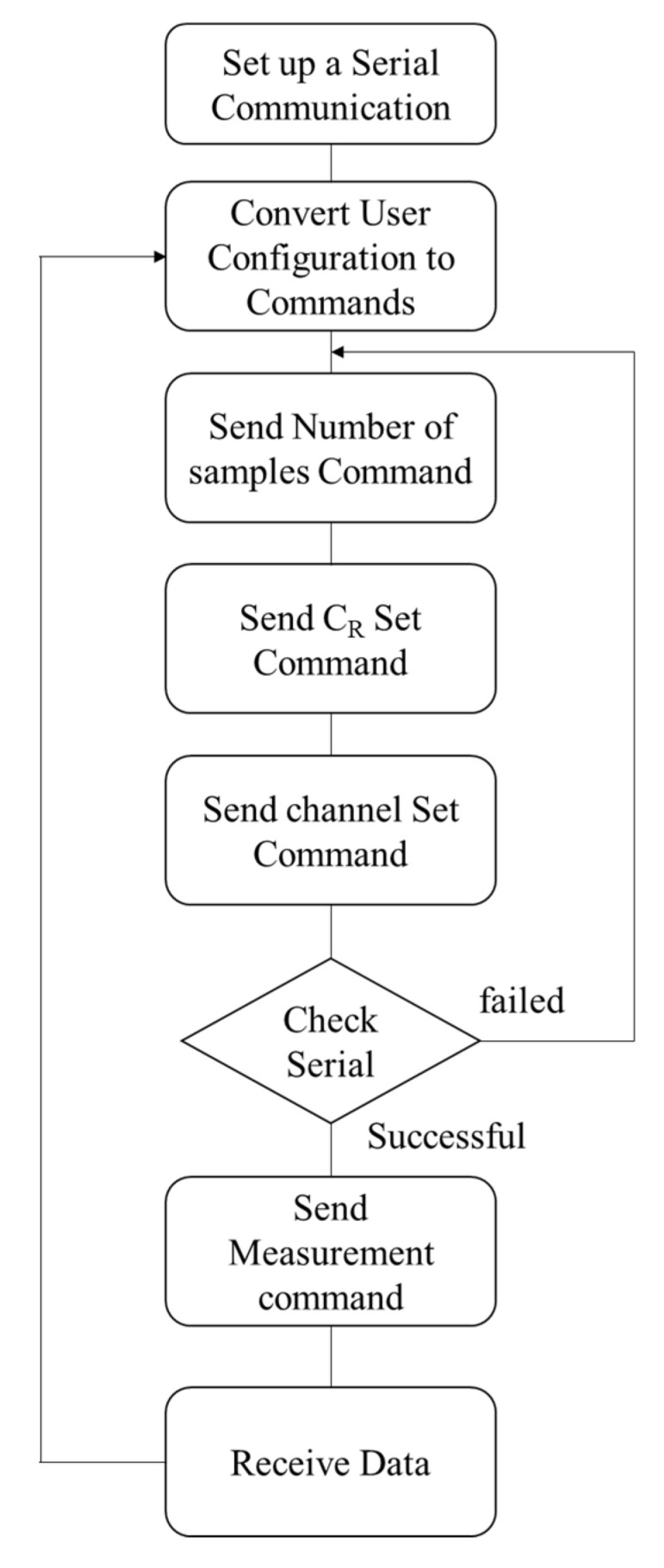
The flowchart of the GUI data acquisition from the Microcontroller: After a serial connection is set up between the GUI and the microcontroller, the user can input all the settings shown in Figure 12a. The GUI generates three commands based on the user settings and sends them to the microcontroller. Then, the serial port is checked again before establishing the measurement command on the serial port. Next, the data is received from the chip and read from the microcontroller’s SPI port buffer.

The real-time visualization and signal processing capability that the GUI provides enables monitoring of the experiment and detection of the time that the sample was introduced as well as the time that the sample evaporates, as shown in Figure 12. Figure 13 illustrates the flowchart of the operation of the GUI that communicates with the microcontroller (Arduino DUE board).

After a serial communication channel has been established between the GUI and the microcontroller unit (MCU), the GUI converts the user input settings to four different types of command, namely the number of samples, and the value of the reference capacitance, and the channel of interest. If writing the commands is successful, the GUI initiates the measurement command. After receiving this command, the MCU transmits the measurement data through the SPI port to the GUI.

#### 3.4.4. Capacitance Calculation

**Figure 14:**
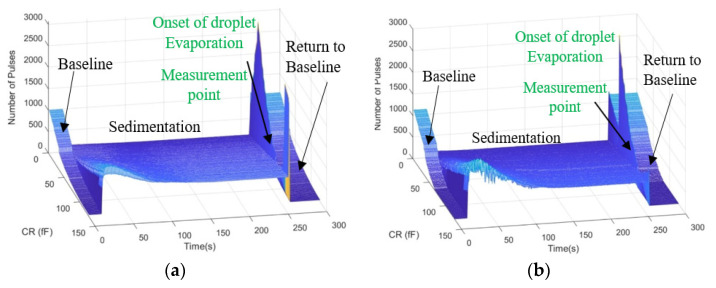
The 3D output of the chip for the first run of the oral samples experiments for (**a**) Left IDE, (**b**) Right IDE.

Figure 14 illustrates the 3D output of the chip for the first run of the experiments with oral cells. The chip’s output is the number of pulses related to the difference in the capacitance seen for the IDEs and the reference capacitance. For a given IDE capacitance, an increase in the reference capacitance results in a decrease in the output number of pulses within the dynamic range of the chip. The sensor’s resolution is about 0.5 fF, which is enough to detect the presence of small oral cells such as neutrophils based on the COMSOL simulation results explained in Section 3.2. Sweeping the bank of capacitors provides a calibration-free capacitance measurement technique, which we previously reported in [40]. The capacitance can be extracted from the 3D curves based on the principle of operation of the sensor. In the differential mode, the sensor’s output will be the same when the sensing and the reference side are in equilibrium. Internal capacitances of 400 fF were added in parallel with the IDEs. A transmission gate on the right circuit enables connecting and disconnecting the IDE to the circuit. The pseudocode given in Algorithm 1 demonstrates the capacitance extraction steps to evaluate the value of the offset capacitance of the IDEs as well as the capacitance change due to the presence of samples.
**Algorithm 1.** Capacitance extraction algorithm from the 3D footprint.1- Switch off the transmission gate and obtain the output curve versus the value of sweeping reference capacitance (number of pulses versus *C*_R_). 2- Calculate the output of the chip for *C*_R_ = 400 fF from the curve obtained in step 1. 3- Turn on the switch and obtain the number of pulses versus the *C*_R_ curve. 4- For the calculated output in step 2, calculate the amount of shift to right. The shifted value is the offset capacitance of the IDE, *C*_IDE_. 5- Calculate the number of pulses for *C*_R_ = 400 + *C*_IDE_ that was obtained in step 4. 6- For the calculated output in step 5, calculate the amount of shift to right after putting the samples. The shifted value is the capacitance increase due to the presence of samples on the electrodes. 7- Repeat the steps for all the next obtained curves to achieve a time-resolved capacitance plot (as shown in Figure 12).

In Figure 14, The sedimentation time refers to when the sample solution still exists on the chip, and as a result, a huge capacitance is created. This capacitance saturates the sensors. Here, saturation is shown by zero in order to differentiate from other measurements.

## 4. Results

In this section, after explaining the fabrication and measurement setup in Section 4.1, the experimental results are demonstrated in Section 4.2.

### 4.1. Fabrication and Measurement Setup

Chip fabrication and its encapsulation are outlined in Section 4.1.1, and next, the whole measurement setup for real-time data acquisition is demonstrated in Section 4.1.2.

#### 4.1.1. Chip Fabrication

**Figure 15:**
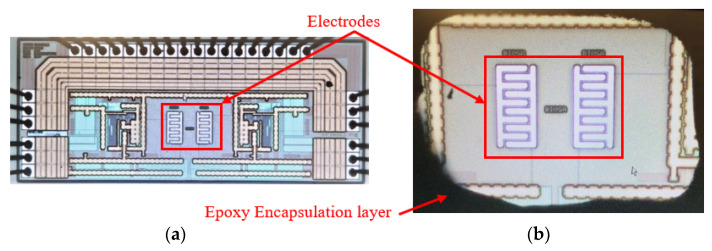
(**a**) Die Micrograph without encapsulation, (**b**) the chamber created after partial encapsulation of the chip.

Our CMOS capacitive chips were fabricated in AMS 0.35 µm high voltage CMOS technology. The chips are composed of two IDEs with five fingers, 12 µm finger width, and pitch, forming an active sensing surface area of 228 µm by 108 µm. Each IDE is connected to a separate differential CDC read-out circuit. Figure 15a represents a die micrograph of the chip. The chip was packaged on a commercial CPGA85 ceramic package with a cavity size of 8.9 mm by 8.9 mm, where the chip occupies a 1 mm × 2 mm area. After packaging, a partial encapsulation was performed on the chip, using a dam and fill technique to cover all the pads on the chip as well as on the package and the bond wires with a non-conductive resin, Hysol CB064/FP4653. After partial encapsulation, a chamber with a rectangular-shaped cross-section with an approximate size of 350 µm by 600 µm was created to serve as the container of the liquid sample, allowing the samples to have direct contact with the two underlying electrodes as depicted in Figure 15b. To be able to distinguish between the electrodes, we call them right and left as they are shown in Figure 15b. There are separate pins for the output of each side.

#### 4.1.2. Measurement Setup

**Figure 16:**
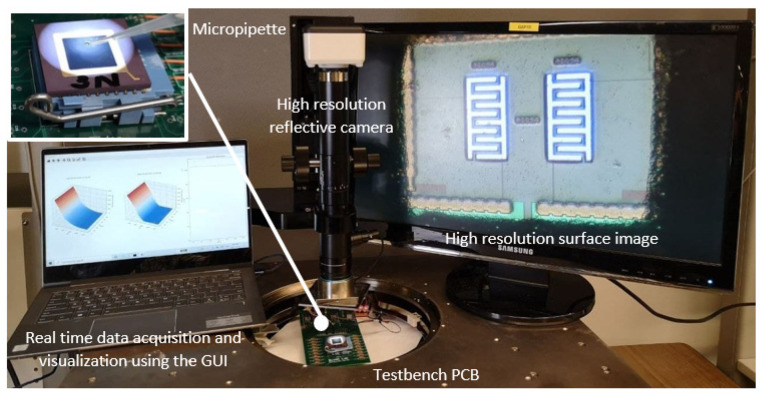
The prepared testbench for real-time data acquisition using the GUI and capturing images before and after cell experiments using the high-resolution reflective camera.

Figure 16 illustrates the testbench prepared for the real-time data acquisition using the GUI and capturing images before and after cell experiments by employing the high-resolution reflective camera. Imaging the surface was challenging for many reflective microscopes due to the partial encapsulation of the chip. We were able to utilize the reflective high-resolution camera of the prob station, TS200-SE. A micropipette was used to manually put 1 µL of oral cell samples on the chip.

### 4.2. Oral Cell-Surface Interaction Results

In this section, the capacitance measurement results are demonstrated in two phases: (1) wet phase and (2) dry phase.

#### 4.2.1. Capacitance Measurement in the Wet Phase

In the beginning, the baseline of each electrode, which is a result of the inherent offset capacitance of the IDE, was evaluated by obtaining the chip’s output without introducing the samples. The output of the chip was obtained for the whole range of values of the bank of capacitors to have a reference point at all possible reference capacitance values. One microliter of each oral sample was placed on the electrodes. The presence of water on the non-passivated chips could increase the capacitance to a saturation level even for the highest value of reference capacitance. As a result, the data obtained before introducing the sample and before water evaporation was considered to determine the cell coverage on the electrodes in the wet mode (see measurement point, Figure 12).

**Figure 17:**
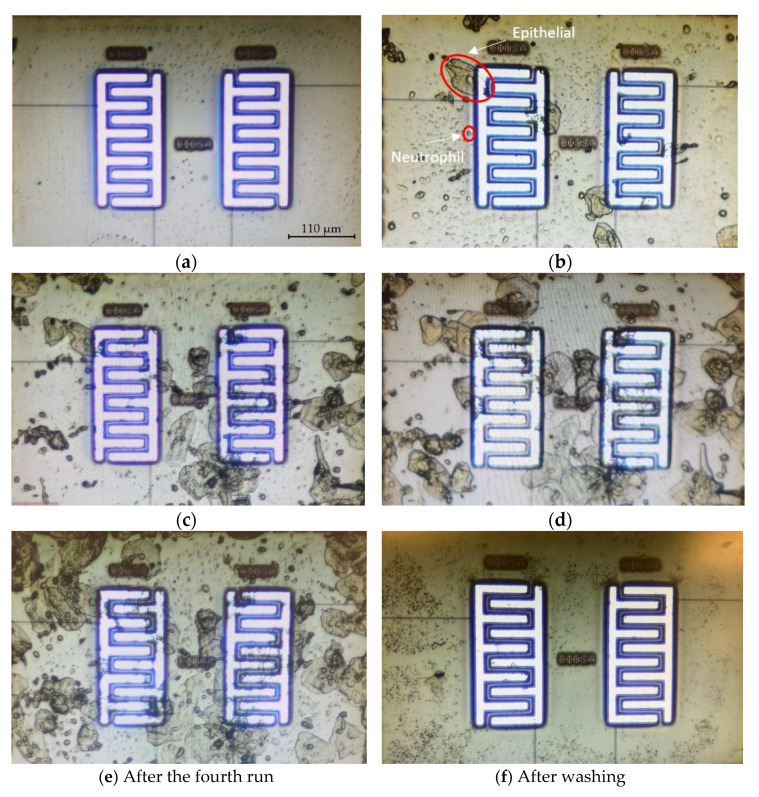
The microscopic image of the surface of the chip for the first experiment: (**a**) before putting samples; (**b**) after the first sample; (**c**) after the second sample; (**d**) after the third sample; (**e**) after the fourth sample; (**f**) after washing (the size of the electrodes is 220 µm × 110 µm, which shows the scale of the images).

Figure 17a illustrates the microscopic image of the surface of the electrodes before the deposition of samples. The deposition of cells, including epithelial and neutrophil cells (shown in Figure 17b) on the electrodes depends upon the hydrodynamic of the evaporation and the tendency of the cells to attach to the surface. Figure 17b shows the microscopic images after the first trial. We put 1 µL of oral sample on the chip and waited until the preliminary water evaporation occurred to eliminate the effect of significant capacitance change (see the Zero value, Figure 12) due to the high volume of water. In Figure 17b, two different types of cells are recognized in the microscopic images. The smaller spherical cells are neutrophils, and the larger cells with nuclei are epithelial cells. Figure 17c–e demonstrate the surface after the second, third, and fourth trials, putting 1 µL of oral sample on the chip and after partial evaporation. It is noteworthy, that the onset of evaporation is where the capacitance sharply drops, as shown in Figure 12. However, this is not the condition that the cells are completely dried.

**Figure 18:**
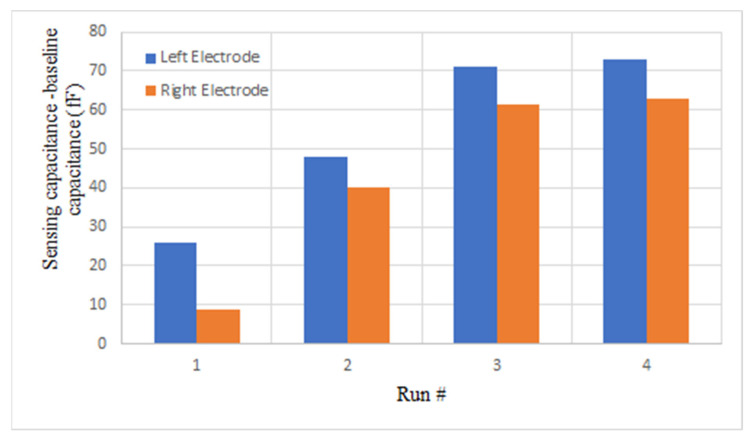
The difference between the capacitance after each run and the capacitance before the first run (fF) (*C*_AfterRun#_-*C*_BeforeRun1_) for four consecutive runs of the first experiment for the two electrodes.

Figure 18 demonstrates the magnitude of capacitance changes after each run with respect to the baseline before the first run (*C*_AfterRun#_-*C*_BeforeRun1_). The results were obtained by performing the capacitance extraction procedure explained in Algorithm 1. By comparing the values of differential capacitance (*C*_AfterRun#_-*C*_BeforeRun#_) and the images shown in Figure 17, there is a correlation between the number of cells that cover the electrode surface and the change in the capacitance. After the first run, there is some coverage of cells on both electrodes, with higher coverage on the left side electrode. The capacitance changes after the first run (*C*_AfterRun1_-*C*_BeforeRun1_) are 26.1 fF and 8.8 fF for the left electrode and the right one, respectively. After the second run, there is more coverage of cells on the right side, and the capacitance change (*C*_AfterRun2_-*C*_BeforeRun2_) is 21.8 fF for the left side, while it is 31.2 fF for the right side. After the third run, cells deposited on the left side have increased as the capacitance change also shows a higher value for the left electrode after the third run. Figure 17f illustrates the microscopic image of the chip after washing, and it shows that the cells were successfully removed with the procedure explained in Section 3.3.2.

#### 4.2.2. Capacitance Measurement in the Dry Phase

**Figure 19:**
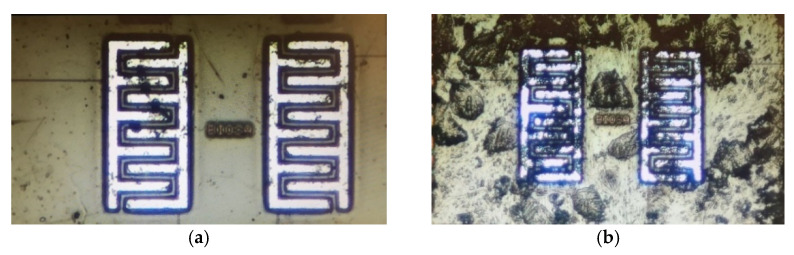

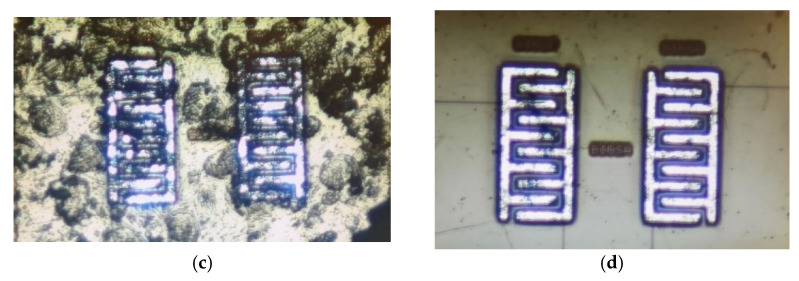
The microscopic images of the surface of the chip for the second experiment: (**a**) After washing and before Run 1; (**b**) After putting the first sample (Run 1); (**c**) After putting the second sample (Run 2); (**d**) The cleaned surface after Run 2.

In the second experiment, the output of the sensor was monitored until the surface completely dried and the output capacitance reached a steady-state value. In the first step, the sensor surface was cleaned with the procedure explained in Section 3.3.2 as shown in Figure 19a. Then, a new prepared sample was introduced to the sensor. The outputs were measured consecutively during two runs without any washing steps in between. Figure 19b,c show the microscopic images of the chip surface after the first and the second runs, respectively. As seen in these figures, the coverage of the chip surface in Figure 19c is more than in Figure 19b. Figure 19d depicts the cleaned chip surface after the second run.

The experimental results almost agree with the COMSOL simulation results presented in Section 3.2. The experimental equivalent capacitance for the sensor in the dry mode when the sensing area is empty and surrounded by air is around 109 fF while the simulation showed a capacitance of around 117 fF for this condition. The small difference between these values can be because of the environmental issues in the real experiment, as well as the differences in the models of CMOS layers used in COMSOL in comparison to the fabricated circuit.

**Figure 20:**
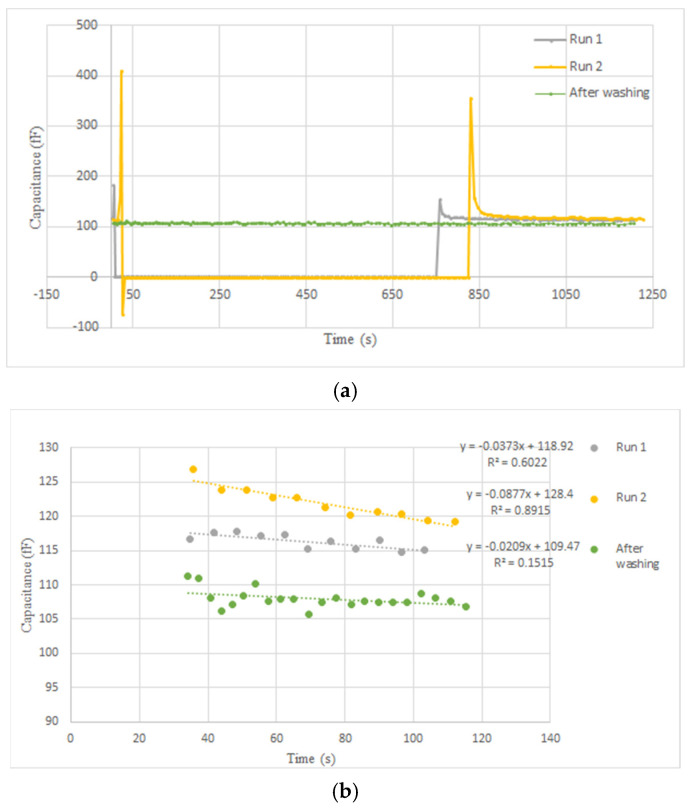
Real-time capacitance measurement result from the right electrode for the second experiment showing: (**a**) the baseline, the saturation state, the transient from the saturation to steady state, as well as the steady state (the saturation state is shown by zero in the figure); (**b**) Steady state (from about 30 s after the peak of the curves depicted in (**a**) for each run).

**Figure 21:**
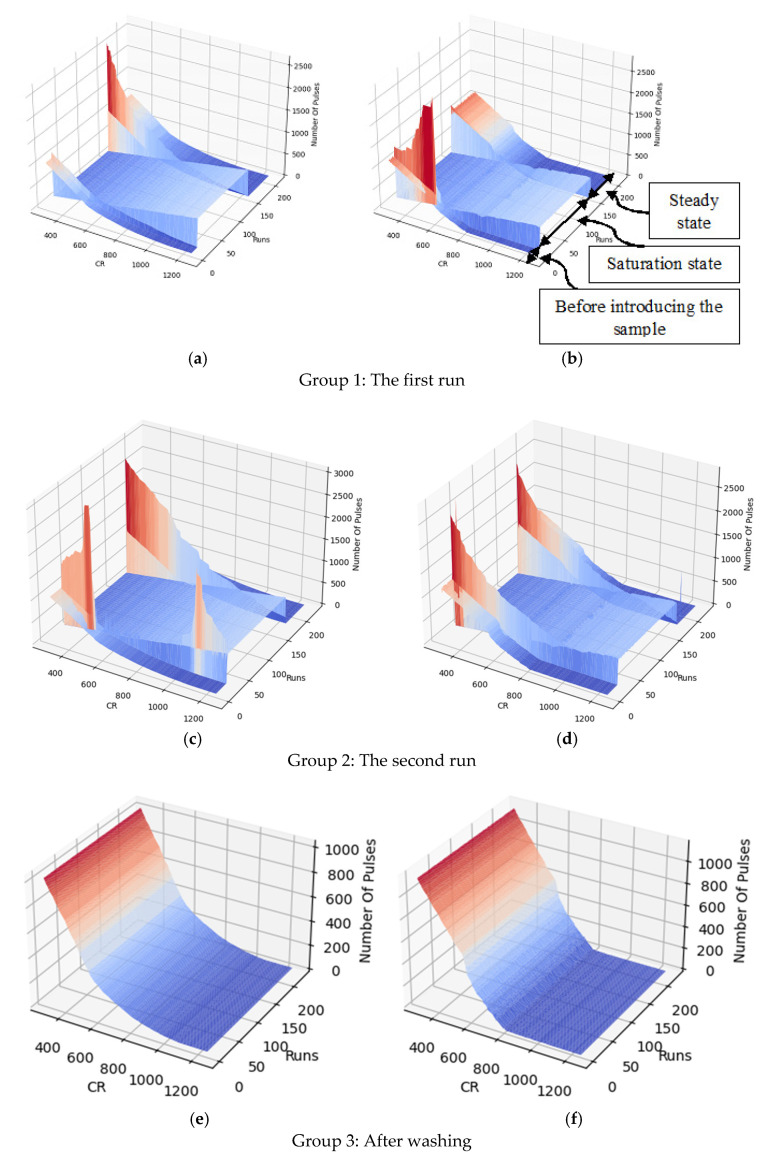
The 3D output of the chip for the second experiment for: (**a**) Run 1, left electrode; (**b**) Run 1, right electrode; (**c**) Run 2, left electrode; (**d**) Run 2, right electrode; (**e**) After washing, left electrode; (**f**) After washing, right electrode.

The capacitance was extracted according to the algorithm explained in Section 3.4.4 Figure 20a,b show the capacitance variations versus time. Real-time measurement of the output during these two trials and after the washing procedure results in three groups of 3D profiles, as shown in Figure 21. Each group includes two 3D profiles for the two electrodes on the left and right.

According to the simulations, the sensor initially gets saturated by introducing the sample and then drops down during the evaporation of the liquid. As shown in Figure 20a and Figure 21, the extracted capacitance and the digital output follow the same trend over time. As aforementioned, the saturation state due to the large capacitance of the liquid is shown by zero in Figure 20a and Figure 21. During the evaporation of the liquid, the output decreases until it reaches a steady-state level.

Figure 20b illustrates the steady states of the two trials and after washing the chip surface. As seen in this figure, adding the new sample to the surface in run 2 without washing after run 1 results in an accumulative output that is higher than the steady-state level of run 1. More coverage of the electrodes by the cells, shown in Figure 19, led to this increasing trend for steady-state capacitance. Washing the surface causes the output to return to the baseline, which is around 109 fF.

## 5. Discussion

In this paper, we introduced a capacitive sensing platform for monitoring oral cells and likely, in the future, analyzing them to understand their links to inflammatory diseases. Herein, we demonstrated the presence of epithelial and neutrophil cells and their effects on capacitive sensors. This takes us one step closer to developing the capacitive sensing PoC device for monitoring and counting the cells. In this direction, there are several practical considerations to be addressed in the future, as discussed below. 

### 5.1. Isolation of Cells from Saliva

Saliva is composed of 99% water and only 1% immunoglobulins, protein, mucus, enzymes, salts, and electrolytes such as sodium, potassium, calcium, magnesium, bicarbonate, and phosphates as well as different types of cells like epithelial cells or intact and lysed inflammatory cells, specifically oPMNs [45,46]. Herein, the standard filtering technique is used to isolate oral cells from saliva. Neutrophils can only be kept alive within a short time frame, and physical techniques are used to purify the sample. To develop a handheld sensing device for monitoring oral neutrophils, which are small, microfluidic techniques are also required to separate them from epithelial cells and the debris in the saliva. The greater the purity of the sample, the greater the accuracy of the measurement result.

### 5.2. The Effects of the Evaporation of the Sample

As explained in Section 4.2, the measurement results depend on the evaporation of the sample. For a reliable measurement, a microfluidic device can be designed to direct the sample toward the sensing site and prevent or control the evaporation of the liquid.

### 5.3. Bubble Creation

**Figure 22:**
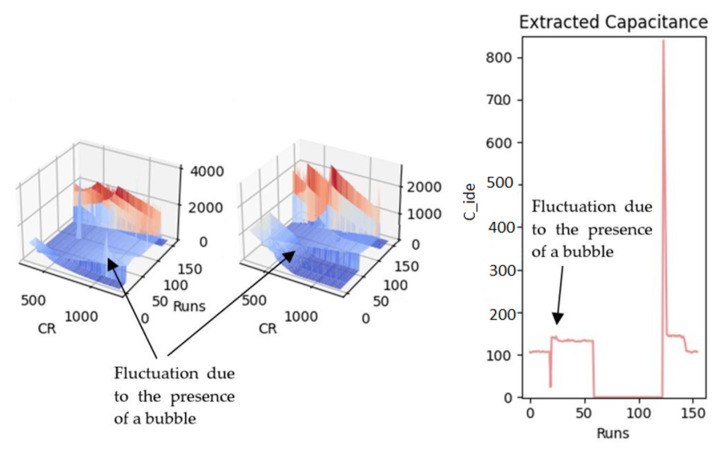
The fluctuated pattern due to the presence of a bubble created during sample placement.

Since pipetting the sample on the chip surface is done manually, some errors are inevitable. For instance, one of the practical problems during the experimental results was the creation of bubbles on top of the sensing area. This phenomenon results in a meaningless output, including fluctuations depending on the bubbles’ presence or absence, as shown in Figure 22. Providing a microfluidic device to control and even automate putting the sample on the chip surface can help avoid bubble formation. In addition, microfluidics makes it possible to put the sample on top of the sensing electrodes more precisely, leading to more accurate measurement results.

### 5.4. Sensing Electrodes

The appropriate location of the cells on top of the sensing area and the amount of cell coverage can affect the measurement results. Since the electrodes do not cover the whole area of the substrate, the cells might be placed over the non-sensitive or less sensitive area, for example in between the electrodes. As a result, the sensor cannot sense them perfectly. To avoid such errors, a new integrated circuit is required whose electrodes cover the whole area of the substrate.

The size of the electrode can also affect its sensitivity. Although we almost cancel the offset in the proposed circuit, the value of the offset and parasitic capacitances might be higher than the sensing capacitance for electrodes that are too large, resulting in a low sensitive measurement. To quantify this, consider that the electrode capacitive ratio (ECR) is defined as α*C* = (∆*C*/*C*_0_) × 100, where ∆*C* is the variations of the sensing capacitance variations due to the presence of the cells and *C*_0_ is the offset and parasitic shares of the electrode–analyte interface. If the size of the electrode is much bigger than the size of the cells, ∆*C* will be much smaller than *C*_0_ leading to a small ECR. On the other hand, although the smaller sizes of the electrodes help increase the ECR, we will need a large number of them to cover the whole area of the substrate so that the circuit can read their outputs in parallel. Herein, we tested a two-electrode sensor with oral cells as a proof of concept. To further our research, we intend to present a new sensor composed of many electrodes for this application.

### 5.5. Read-Out Circuit Specifications

Various factors such as parasitic effects, systematic errors, environmental factors, and experiment-time offset variation due to the remnants of the cells can create a time-variant offset capacitance that might saturate the sensor’s output. Therefore, the sensor should have a wide IDR to show the target cells’ concentration. According to Table 1, the proposed sensor in this work offers a wider IDR than the other reported capacitive sensors. In addition, the IDR of the sensor is programmable, and the employed calibration-free technique is based on sweeping the reference capacitor [40]. This has paved the way to mitigate the effects of undesired time-variant offsets significantly.

On the other hand, a higher sensor resolution can lead to more accuracy. If the cell is not completely located on the electrode, the sensor can detect the part of the cell covering the electrode. There is a trade-off between IDR and the resolution of a circuit.

Raising the gain of current amplifiers and the sensitivity of the CCO can help to improve the sensitivity of the sensor. Increasing the IDR of the CCO and the size of the counter can also result in a wider IDR. These topics are deferred to future work.

## 6. Conclusions

This paper demonstrated the applicability of a calibration-free and wide-IDR CMOS capacitive sensor for monitoring oral cells in saliva samples. The capacitance of the on-chip IDEs was mathematically modeled by considering the existing parasitic and fringe capacitances. A COMSOL simulation was performed to qualitatively gain insight from the sensor response to the biological cells. Simulations were qualitatively in agreement with the experiment and confirmed the functionality of devices in terms of sensitivity to change in the dielectric of added material on its sensing surface. The difference between the capacitance values before and after water evaporation could represent the amount of electrode surface coverage by oral cells. By taking advantage of the wide IDR of the sensor and the programmable bank of reference capacitors, the accumulation of the cells could be assessed over four runs without washing. To avoid the electrodes’ conditions being manipulated after each run, no washing steps were applied to the electrodes exposed to the oral cells sample. The experiments showed that the accumulation of the cells after each run decreases the sensor’s sensitivity. Therefore, a cleaning procedure was employed to remove the cells from the chip surface. The results were encouraging regarding developing reusable, integrated sensing devices that can set the stage for quantifying and analyzing oral cells, including neutrophils and epithelial cells, in the future.

## Figures and Tables

**Table 1:** Comparison of the capacitive sensors reported for cellular applications.

CMOS Technology	Type of Cell	Electrode Material	Resolution	IDR (fF)	Reference
0.5 µm	hBC	Al/1pass	5 fF	NA	[34]
0.35 µm	hOC	Al/SiO_2_/Si_3_N_4_	14.4 aF	12	[21,35,42]
0.25 µm	*S. epidermidis*	Al/Al_2_O_3_	10 fF	NA	[37]
0.25 µm	*S. epidermidis*	Al/Al_2_O_3_	450 aF	57	[38]
0.18 µm	*E.Coli*	Al/wPass	10 aF	2.7	[23]
0.35 µm	hLC	Al/PEM	10 aF	10	[22,43]
90 nm	hBC	AuCu	<10 aF	NA	[25,36]
0.35 µm	Oral cells	Al/A_2_O_3_	416 aF	400	This work

IDR: Input Dynamic Range, hBC: Human Breast Cancer cell, hOC: Human Ovarian Cancer cell, hLC: Human Lung Carcinoma cell, Al/1pass: Al electrode with One passivation layer, Al/wPass: Passivated Aluminum interdigitated electrode with a window in between the fingers, PEM: Polyelectrolyte Multilayer.

**Figure A1:**
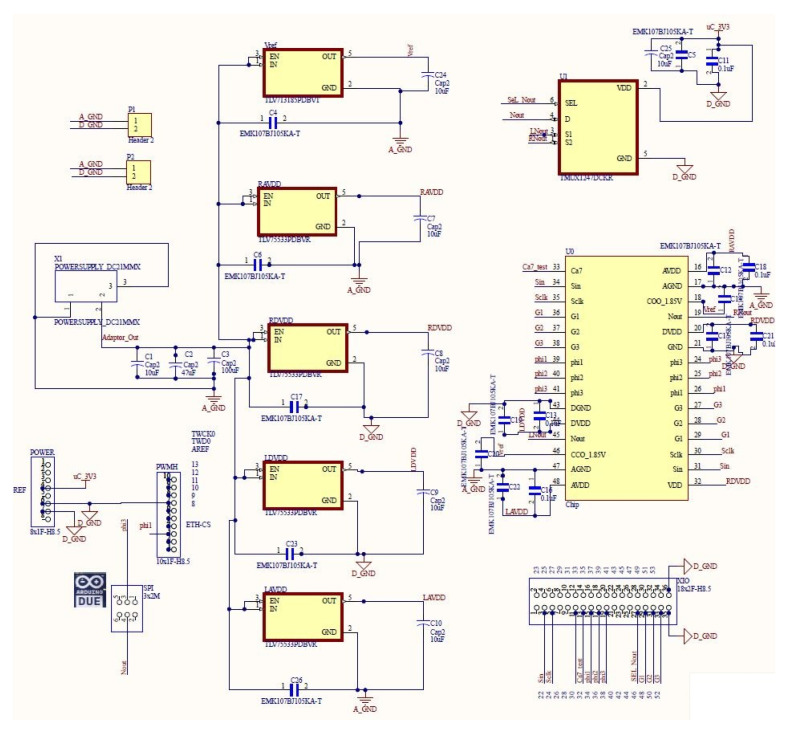
The schematic of the PCB of the sensor shown in Figure 9.

## Data Availability

The data are not publicly available.

## Appendix A

Figure A1 illustrates the schematic of the PCB of the sensor shown in Figure 9. In this figure, the block shown with U0 represents the chip while U1 represents the 2:1 multiplexer chip that is implemented to switch between right and left channels. Vref is a voltage regulator block of 1.85 V output that is utilized for the CCO block. RAVDD, RDVDD, LAVDD, and LDVDD blocks are 3.3 V-output voltage regulators that provide the VDD voltage to the right analog block, right digital block, left analog block, and left digital block, respectively. The input is an external 5 V DC source shown as X1. Connectors are for the Arduino DUE board, making the digital interface between the board and Arduino. Multiple decoupling capacitors are used between voltage and ground rails.
